# Supplementary material for: Hybrid Models and Biological Model Reduction with PyDSTool
Source: PLoS Comput Biol. 2012 Aug 9;8(8):e1002628. doi: 10.1371/journal.pcbi.1002628 (PMC3415397; doi:10.1371/journal.pcbi.1002628)
Supplement: Text S4 — Complete source code for the PyDSTool package (version 0.88.120504). Includes API documentation and help files linking to web pages. This file is identical to the current public release on Sourceforge.net. (ZIP) [file pcbi.1002628.s004.zip › PyDSTool/html/toc.html]

xml version="1.0" encoding="ascii"?


Table of Contents


# Table of Contents

---

Everything
  

## Modules

PyDSTool  
 PyDSTool.Events  
 PyDSTool.FuncSpec'  
 PyDSTool.Generator  
 PyDSTool.Generator.ADMC\_ODEsystem'  
 PyDSTool.Generator.DDEsystem  
 PyDSTool.Generator.Dopri\_ODEsystem'  
 PyDSTool.Generator.EmbeddedSysGen'  
 PyDSTool.Generator.Euler\_ODEsystem'  
 PyDSTool.Generator.ExplicitFnGen'  
 PyDSTool.Generator.ExtrapolateTable'  
 PyDSTool.Generator.ImplicitFnGen'  
 PyDSTool.Generator.InterpolateTable'  
 PyDSTool.Generator.LookupTable'  
 PyDSTool.Generator.MapSystem'  
 PyDSTool.Generator.ODEsystem'  
 PyDSTool.Generator.Radau\_ODEsystem'  
 PyDSTool.Generator.Vode\_ODEsystem'  
 PyDSTool.Generator.allimports  
 PyDSTool.Generator.baseclasses  
 PyDSTool.Generator.messagecodes  
 PyDSTool.Interval'  
 PyDSTool.MProject  
 PyDSTool.Model  
 PyDSTool.ModelConstructor'  
 PyDSTool.ModelSpec'  
 PyDSTool.Points  
 PyDSTool.PyCont  
 PyDSTool.PyCont.BifPoint  
 PyDSTool.PyCont.ContClass'  
 PyDSTool.PyCont.Continuation  
 PyDSTool.PyCont.Plotting  
 PyDSTool.PyCont.TestFunc  
 PyDSTool.PyCont.misc  
 PyDSTool.Redirector  
 PyDSTool.Symbolic  
 PyDSTool.Toolbox  
 PyDSTool.Toolbox.ActivationFuncs  
 PyDSTool.Toolbox.DSSRT\_tools  
 PyDSTool.Toolbox.FR  
 PyDSTool.Toolbox.FSM  
 PyDSTool.Toolbox.InputProfile  
 PyDSTool.Toolbox.ModelEst  
 PyDSTool.Toolbox.ModelHelper  
 PyDSTool.Toolbox.NineML  
 PyDSTool.Toolbox.PRCtools  
 PyDSTool.Toolbox.ParamEst  
 PyDSTool.Toolbox.PySCes\_SBML  
 PyDSTool.Toolbox.adjointPRC  
 PyDSTool.Toolbox.data\_analysis  
 PyDSTool.Toolbox.dataanalysis  
 PyDSTool.Toolbox.dssrt  
 PyDSTool.Toolbox.event\_driven\_simulator  
 PyDSTool.Toolbox.fracdim  
 PyDSTool.Toolbox.makeSloppyModel  
 PyDSTool.Toolbox.mechmatlib  
 PyDSTool.Toolbox.model\_primitives  
 PyDSTool.Toolbox.neuralcomp  
 PyDSTool.Toolbox.neuro\_data  
 PyDSTool.Toolbox.optimizers  
 PyDSTool.Toolbox.optimizers.criterion  

PyDSTool.Toolbox.optimizers.criterion.composite\_criteria

PyDSTool.Toolbox.optimizers.criterion.criteria

PyDSTool.Toolbox.optimizers.criterion.facilities

PyDSTool.Toolbox.optimizers.criterion.information\_criteria

PyDSTool.Toolbox.optimizers.defaults  
 PyDSTool.Toolbox.optimizers.helpers  

PyDSTool.Toolbox.optimizers.helpers.finite\_difference

PyDSTool.Toolbox.optimizers.helpers.levenberg\_marquardt  

PyDSTool.Toolbox.optimizers.helpers.quadratic

PyDSTool.Toolbox.optimizers.line\_search  

PyDSTool.Toolbox.optimizers.line\_search.adaptive\_last\_step\_modifier

PyDSTool.Toolbox.optimizers.line\_search.backtracking\_search

PyDSTool.Toolbox.optimizers.line\_search.barzilai\_borwein\_non\_monotone\_search  
 PyDSTool.Toolbox.optimizers.line\_search.barzilai\_borwein\_search  

PyDSTool.Toolbox.optimizers.line\_search.cubic\_interpolation

PyDSTool.Toolbox.optimizers.line\_search.damped\_line\_search

PyDSTool.Toolbox.optimizers.line\_search.fibonacci\_section

PyDSTool.Toolbox.optimizers.line\_search.fixed\_last\_step\_modifier

PyDSTool.Toolbox.optimizers.line\_search.golden\_section

PyDSTool.Toolbox.optimizers.line\_search.goldstein\_rule

PyDSTool.Toolbox.optimizers.line\_search.hyperbolic\_line\_search

PyDSTool.Toolbox.optimizers.line\_search.quadratic\_interpolation

PyDSTool.Toolbox.optimizers.line\_search.scaled\_line\_search

PyDSTool.Toolbox.optimizers.line\_search.simple\_line\_search

PyDSTool.Toolbox.optimizers.line\_search.strong\_wolfe\_powell\_rule

PyDSTool.Toolbox.optimizers.line\_search.wolfe\_powell\_rule

PyDSTool.Toolbox.optimizers.optimizer  

PyDSTool.Toolbox.optimizers.optimizer.optimizer

PyDSTool.Toolbox.optimizers.optimizer.standard\_optimizer

PyDSTool.Toolbox.optimizers.optimizer.standard\_optimizer\_modifying

PyDSTool.Toolbox.optimizers.step  

PyDSTool.Toolbox.optimizers.step.conjugate\_gradient\_step

PyDSTool.Toolbox.optimizers.step.goldfeld\_step

PyDSTool.Toolbox.optimizers.step.goldstein\_price\_step

PyDSTool.Toolbox.optimizers.step.gradient\_step

PyDSTool.Toolbox.optimizers.step.local\_brute\_force\_1dstep

PyDSTool.Toolbox.optimizers.step.marquardt\_step

PyDSTool.Toolbox.optimizers.step.newton\_step

PyDSTool.Toolbox.optimizers.step.partial\_step

PyDSTool.Toolbox.optimizers.step.quasi\_newton\_step

PyDSTool.Toolbox.optimizers.step.restart\_conjugate\_gradient

PyDSTool.Toolbox.optimizers.tests  
 PyDSTool.Toolbox.optimizers.tests.test\_powell  
 PyDSTool.Toolbox.optimizers.tests.test\_quadratic  
 PyDSTool.Toolbox.optimizers.tests.test\_rosenbrock  
 PyDSTool.Toolbox.phaseplane  
 PyDSTool.Toolbox.prep\_boxplot  
 PyDSTool.Toolbox.synthetic\_data  
 PyDSTool.Toolbox.syntheticdata  
 PyDSTool.Toolbox.test\_protocols  
 PyDSTool.Trajectory'  
 PyDSTool.Variable'  
 PyDSTool.common  
 PyDSTool.conf  
 PyDSTool.errors  
 PyDSTool.fixedpickle  
 PyDSTool.integrator'  
 PyDSTool.matplotlib\_import  
 PyDSTool.parseUtils  
 PyDSTool.scipy\_ode  
 PyDSTool.utils  
 cPickle  
 matplotlib.pylab  
 scipy.integrate.vode  

---

[hide private]
